# Editorial: Advances in the use of EGFR TKIs in the treatment of NSCLC

**DOI:** 10.3389/fonc.2024.1506160

**Published:** 2024-10-31

**Authors:** Paola Ulivi

**Affiliations:** Bioscience Laboratory, IRCCS Istituto Romagnolo per lo Studio dei Tumori (IRST) “Dino Amadori”, Meldola, Italy

**Keywords:** EGFR, TKI, lung cancer, liquid biopsy, resistance

Lung cancer is the leading cause of cancer-related deaths worldwide. Non-small cell lung cancer (NSCLC) is the most common type of lung cancer, accounting for approximately 85% of all cases. Over the last decade, there have been significant advances in the treatment of NSCLC, particularly with the use of epidermal growth factor receptor (EGFR) tyrosine kinase inhibitors (TKIs). EGFR is a key driver of NSCLC, and EGFR TKIs have shown remarkable clinical activity in patients with EGFR-mutant NSCLC.

Despite these advances, however, there are still many unanswered questions regarding the optimal use of EGFR TKIs in the treatment of NSCLC. To improve patient outcomes, further research into the use of EGFR TKIs in lung cancer therapy is vital.

This Research Topic collected 20 publications: nine original articles, seven case reports, two reviews, one meta-analysis and one leader opinion.

Several aspects of EGFR TKI treatment have been discussed.

The issue of TKIs resistance has been treated in some papers. In their Opinion, Bronte et al. discussed the topic of Osimertinib resistance and the possible combination strategies or the use of fourth generation EGFR-TKIs to overcome it. They supposed that in the next future oncologists will be able to address each patients who experience resistance to upfront osimertinib toward a different treatment strategy on the basis of molecular alterations highlighted by liquid biopsy or tumor biopsy. In this line, Carlo Bao et al. presented two clinical cases harboring concomitant EGFR and BRAF alterations and treated with Osimertinib. Both cases were responsive to treatment and liquid biopsy results were in accordance with the clinical behavior. Patients with advanced NSCLC with secondary T790M can benefit from Osimertinib, but the role of this drug in patients who exhibit resistance without T790M or with T790M unknown status is not well established. From the systematic review and meta-analysis performed by Yi et al. emerged that patients who undergo progression with brain metastasis on first or second generation TKIs can benefit from subsequent Osimertinib regardless T790M status. This represent an important finding that could orientate clinical decision management after Osimertinib resistance.

Other types of resistance mechanisms are known, and the role of tumor-associated macrophages (TAMs) is also emerging. Previous studies have demonstrated that high infiltration of TAMs is significantly associated with an unfavorable prognosis in NSCLC patients treated with EGFR-TKIs (1). So, the inhibition of TAMs may a potential approach to improving resistance to EGFR-TKIs. In the review reported by Cheng et al., a number of preclinical studies have been discussed, analyzing the combination of EGFR-TKIs with several compound directed versus TAMs. In particular, inhibiting mTOR, AKT and STAT3 pathways, such as the lipid metabolic pathway, could all be possible potential strategies to overcome EGFR-TKI resistance.

The addition of antiangiogenic treatments to EGFR-TKis was also studied. The systematic review and meta-analysis by Zheng et al. highlighted that the addition of bevacizumab to EGFR-TKI provide significant better PFS and OS, and the benefit is more evident for patients who have ever smoked, aged <75 years and of Asian population. Hsu et al. reported the results of a study in which first-line bevacizumab was combined with erlotinib or afatinib in EGFR-mutated NSCLC, demonstrating the development of T790M as resistance mechanism in 57.9% of cases. The addition of anti-angiogenic treatment in patients with an acquired resistance to Osimertinib was also demonstrated in a clinical case described by He et al. In particular, a NSCLC patients harboring acquired EGFR 19Del/T790M/cis-C797S mutation resistance was treated with sintilimab, an anti-VEGF drug and chemotherapy. The patient remained progression-free for 15 months and the regimen was well tolerated. The addition of immunotherapy in EGFR-mutated patients is a controversial topic (2). Microsatellite instability is a rare event in NSCLC, but Yang et al. reported a case of a patient with a rare pulmonary enteric adenocarcinoma with EGFR mutation and MSI-H, who receive benefit from a combination approach of EGFR-TKI plus immune check point inhibitor.

Rare EGFR mutations are another important issue object of several studies in the last years. It was reported that afatinib, an irreversible ErbB family inhibitor, is more efficacious in treating patients with uncommon EGFR mutations (3).

Dong et al. performed a literature search on studies evaluating the efficacy of afatinib in any line of treatment in NSCLC patients with uncommon EGFR mutations. Their conclusion was that afatinib seems to be effective in patients with the more frequent uncommon EGFR mutations, whereas inconsistent data are present with regard to the other uncommon EGFR alterations, due to the high heterogeneity of them. Christopoulos et al. described seven clinical cases of patients carrying different uncommon EGFR mutations treated with afatinib. Overall, patients responded to afatinib, with six partial response and three durable responses. Interestingly, co-mutations did not preclude sensitivity to the drug, with durable response observed also in patients exhibiting co-occurring TP53 or CDKN2A mutations. Another case report was reported by Wang et al., describing a patient carrying two exon 18 mutations and who acquired an EGFR amplification after treatment with osimertinib. The patient showed a partial response to neratinib. Also, a further case report of a patient carrying an EGFR kinase domain duplication was reported by Lin et al. The patient received the 3rd generation EGFR-TKI furmonertinib and obtained a partial response in primary tumor and in central nervous system metastases. The efficacy of Furmonertinib was also studied in a real word setting in the study of Yan et al. The authors analyzed a cohort of EGFR mutated patients treated with furmonertinib as first line treatment. They observed a median PFS of 19.5 months without significant correlations with ECOG, presence of brain or livera metastasis, sex, age EGFR status or number of metastatic sites. The author suggested that the use of furmonertinib could be a valid option for the first line treatment of EGFR mutated patients, also considering the manageable nature of adverse events. Furmonertinib was also studied specifically in EGFR exon 20 insertion positive NSCLC in the study of Hu et al. Patients were treated in the first line setting and the median PFS observed was of 7.2 months, with a good safety profile.

Although adenocarcinoma is the predominat NSCLC histotype carrying EGFR mutation, also adenosquamous cell carcinoma, a low incidence histotype, could in some cases carry an EGFR mutation (4). Xia et al. explored the efficacy of EGFR-TKis. They observed an efficacy of EGFR-TKIs similar to that observed in adenocarcinoma.

EGFR-TKIs are also used in the non-metastatic disease. In the neo-adjuvant setting Shao et al. conducted a small study with the aim to compare the efficacy of neoadjuvant targeted therapy versus targeted combined with chemotherapy in operable EGFR-mutated NSCLC, concluding that targeted therapy alone was equally effective and more safety with respect to the combination regimen. In the adjuvant setting, there is a debatable question about the treatment of patients at recurrence after adjuvant osimertinib. An international Delphi consensus report was published in the paper by Mirza et al., reporting the conclusions of a panel of experts after discussing of this topics. Consensus was reached on six statements describing treatment considerations for the specific NSCLC recurrence scenarios, and agreed that more clinical trials are required before precise recommendations for specific patient populations can be made.

From a biological point of view, the identification of biomarkers able to give prognostic and predictive indications represent a crucial point in cancer. EGFR-mutated NSCLC could have different prognosis and different response to targeted treatment. Secretome is represented by proteins released from tumor cells that could regulate several pathways involved in cancer proliferation. In NSCLC recent studies have identified specific proteins affecting TKI resistance. Luu et al. Performed a mass spectrometry analysis on the secretome of EGFR mutated cells representing different stages of NSCC transformation, and identified three candidates (MDK, GDF15, SPINT2) associated with a poor survival.

Discovery biological studies are needed to identify novel potential biomarkers in NSCLC. Abudereheman et al. reported a study in which RNASequencing and Whole Exome Sequencing results were examined in patients with tuberculosis, with the aim to examine and identify crucial genes implicated in NSCLC genesis. They identified four genes (EGFR, CECR2, LAMA3 and HSPA2) that play an important role in lung cancer tumorigenesis.

Other than EGFR-TKIs, other targeted treatment are both approved in the clinical practice and also under investigation. A clinical case of a patient with adenocarcinoma and malignant pleural effusion, carrying a ROS1 rearrangement, was reported by Tian et al. The patient was treated with Crizotinib and Anlotinib, with a significant reduction and even disappearance of the malignant effusion, suggesting that this drug combination could be a promising strategy for the treatment of ROS1 rearranged tumors.
